# A simple twist technique for lens-sparing one-handed peripheral vitrectomy in phakic patients: a learning approach for junior surgeons

**DOI:** 10.1186/s40942-022-00433-w

**Published:** 2022-12-12

**Authors:** Goran Petrovski, Lyubomyr Lytvynchuk, Knut Stieger, David Petrovski

**Affiliations:** 1 Center for Eye Research and Innovative Diagnostics, Department of Ophthalmology, Institute for Clinical Medicine, Faculty of Medicine, Oslo University Hospital, University of Oslo, Oslo, Norway; 2 Department of Ophthalmology, University of Split School of Medicine and University Hospital Centre, 21000 Split, Croatia; 3 Department of Ophthalmology, Justus-Liebig-University Giessen, University Hospital Giessen and Marburg GmbH, Giessen, Germany; 4 Elvebakken VGS, Oslo, Norway

**Keywords:** Lens-sparing, Twist technique, One-handed vitrectomy

## Abstract

The evolution of vitrectomy has led to improved suturless techniques and minimally invasive surgery. Nevertheless, the procedure requires great bimanual dexterity and poses risk for lens touch, especially in the hands of less experienced junior surgeons. We hereby present a twist technique which allows for one-handed (right or left) peripheral vitrectomy without the need for one or several hand-switches with the vitreous cutter and avoids lens touch. The technique can be used as a learning approach for junior vitreoretinal surgeons.

**Supplementary Information:**

The online version contains supplementary material available at 10.1186/s40942-022-00433-w.

## Background

Since the introduction of vitrectomy by Dr. Robert Machemer in the 1970s (Machemer 1972), the surgical technique has evolved from a low to high and ultrahigh cut rate, with improved fluidics also coming into place. Meanwhile, the sclerotomy sites have become smaller and self-sealing, the sutureless technique being commonly applied nowadays using 23G, 25G, 27G, and even 29G and 30G trocars. transconjunctivally (Chang 2008).

In parallel to the improvements in the size of the sclerotomies and the instrumental design of the trocars/vitrectomy probes, the vitrectomy as a technique has also evolved, with simplier and safer surgery being practiced in mostly day-surgery setting.

On the side of the patient, having a clear lens or being phakic, vitrectomy has not changed much over the years as being the cause of cataract development or its advancement, while lens touch has been the unwanted side-effect of surgery *per se*, in particular during the less experienced days of the learning curve in vitreoretinal surgery. Recent studies on the prevalence of lens touch have reported a 1.2–3.7% of phakic patients experiencing such complications of surgery (Elhousseini 2016; Jackson 2013). Although these complications are more often seen in patients with retinal detachment having proliferative vitreoretinopathy, equally important and prevalent is the group of phakic patients undergoing vitrectomy due to maculopathy (epiretinal fibrosis, macular hole, vitreomacular traction syndrome). A technique of inducing intentional continuous shallowing of the anterior chamber has been previously suggested to prevent lens touch (Mulder 2015).

The lens of a phakic patient can be injured during placement of the trocars, as well as moving of the vitrectomy-, endoillumination- or endolaser- probes in and out of the eye (Additional file 1: Video S1). In phakic patients, the trocars are usually placed 4 mm posterior to the limbus, with careful attention being paid to the angle of scleral incision into the globe. The position of the light and vitrectomy trocars is usually between the 9:30 − 11:00 and 1:00–2:30 clock-hours. A great bimanual dexterity is required during the procedure, which is difficult for junior vitreoretinal surgens climbing the learning curve. Some surgeons prefer having an air bubble enter the vitreous cavity through the infusion line, so to mark the border of the posterior surface of the lens and avoid lens touch. A rule of thumb in doing vitrectomy on phacic patients has been not to cross the midline with the vitreous cutter. Under such conditions, however, performing a one-handed or no-hand-switch peripheral vitrectomy would not be possible (Additional file 1: Video S1).

## Description of the twist technique

**Fig. 1:**
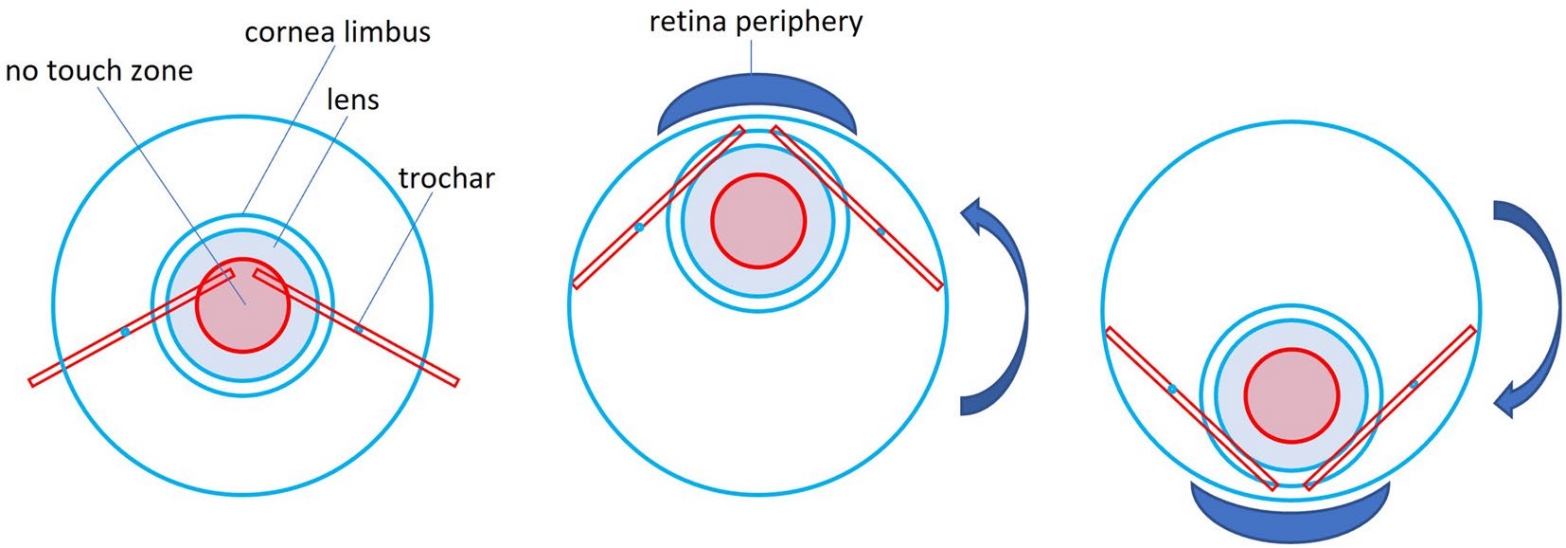
Schematic of the lens-sparing twist technique bypassing the “No touch zone” of the lens

We hereby present a twist technique which allows for one-handed (right or left) peripheral vitrectomy without the need for one or several hand-switches with the vitreous cutter (Additional file 1: Video S1). The twist technique, even though it does not change the the relative position between the trocars and the lens, it allows to bypass the “No touch zone” of the lens, where it is deepest posteriorly (Fig. 1).

The twist technique for a right-handed surgeon or a right-handed vitrectomy entrance on a right eye would imply the use of intorsion—a down- and tilting-in movement of the eye which allows peripheral vitrectomy in the 3–6 o’clock area, and extorsion- an up- and tilting-in movement of the eye which allows peripheral vitrectomy in the 12 − 3 o’clock area. The technique requires the working trocars to be positioned between the 9:00–9:30 and 2:30−3:00 clock hours. An animation and a video of the surgical procedure using the proposed movements during vitrectomy under the same conditions are shown in Additional file 1: Videos S1 and Additional file 2: Video S2, respectively, as well as phantom surgery in Videos Additional file 3:Video S3 (showing both, a right- and left-handed approach; EYESI Simulator Platform 2.6, VRmagic GmbH, Mannheim, Germany). The case presented in Additional file 2: Video S2 maintained a clear lens throughout the whole right-handed surgery involving peeling of epiretinal fibrosis and internal limiting membrane, as well as peeling of a fibrovascular proliferative membrane over the optic disc and pan-retinal photocoagulation, all being performed using the one-handed twist technique.

## Summary

Lens-sparing minimally invasive vitrectomy approach remains a method of choise to treat localized retinal disorders, such as epiretinal membranes, macular holes and vitreomacular traction syndrome. However, an unintentional intraoperative lens injury can lead to postoperative developement of iatrogenic cataract; which could require additional surgery. Doing a simple twist technique in a one-handed manner to achieve peripheral vitrectomy in phakic patients is a simple and easy-tp-perform approach during the learning stages for junior surgeons and beyond, allowing to decrease the risk of intraoperative lens touch.

## Supplementary Information

**Additional file 1: Video S1.** Animated presentation of the lens-sparing twist technique movement during a right-handed vitrectomy.

**Additional file 2: Video S2.** Animated actual vitrectomy procedure showing the extorsion and intorsion movements int he lens-sparing twist technique and persistence of clear lens throughout surgery.

**Additional file 3: Video S3.** Phantom surgery showing the lens-sparing twist technique for a right-handed and a left-handed surgery. Lens touch is also shown. (EYESI Simulator Platform 2.6, VRmagic GmbH, Mannheim, Germany).

## Data Availability

All data will be provided upon request from the corresponding author.
